# Prognostic and predictive value of clinical and biochemical factors in breast cancer patients with bone metastases receiving "metronomic" zoledronic acid

**DOI:** 10.1186/1471-2407-11-403

**Published:** 2011-09-22

**Authors:** Xinmin Zhao, Xiaofeng Xu, Qunling Zhang, Zhen Jia, Si Sun, Jian Zhang, Biyun Wang, Zhonghua Wang, Xichun Hu

**Affiliations:** 1 Department of Medical Oncology, Fudan University Shanghai Cancer Center; Department of Oncology, Shanghai Medical College, Fudan University, No. 270, Dong An Road, Shanghai 200032, China; 2 Department of Clinical Laboratory, Fudan University Shanghai Cancer Center; Department of Oncology, Shanghai Medical College, Fudan University, No. 270, Dong An Road, Shanghai 200032, China

**Keywords:** Advanced breast cancer, bone metastases, zoledronic acid, VEGF, N-telopeptide, prognosis, predictive factors

## Abstract

**Background:**

To assess prognostic and predictive effects of clinical and biochemical factors in our published randomized study of a weekly low dose (metronomic arm) versus a conventional dosage of zoledronic acid (conventional arm) in breast cancer patients with bone metastases.

**Methods:**

Treatment outcome of 60 patients with bone metastases were used to assess impacts of following potential prognostic factors, estrogen receptor status, lymph node status, 2 year-disease free interval (DFI), numbers of chemotherapy regimens administered, interventions, and serum levels of VEGF, N-telopeptide of type I collagen (NTx), CEA, and CA 15-3.

**Results:**

In univariate analyses, patients pretreated with 2 or fewer chemotherapy regimens, ER-positive tumors, 3 or fewer lymph nodes, DFI of more than 2 years, serum VEGF of less than 500 pg/mL after 3 months of intervention, serum CEA and CA 15-3 of less than ULN, and baseline serum NTx of less than 18 nM BCE had significantly longer progression free survival (PFS). The multivariate analysis showed that ER positivity (hazard ratio [HR], 0.295; 95% confidence interval [CI], 0.141-0.618; P = 0.001), serum VEGF of less than 500 pg/mL after 3 months of intervention (HR, 2.220; 95% CI, 1.136-4.338; P = 0.020), baseline serum NTx of less than 18 nM BCE (HR, 2.842; 95% CI, 1.458-5.539; P = 0.001), and 2 or fewer chemotherapy regimens received (HR, 7.803; 95% CI, 2.884-21.112; P = 0.000) were associated with a better PFS. When evaluating the predictive effect of the biochemical factors, an interaction between NTx and zoledronic acid intervention was shown (P = 0.005). The HR of weekly low dose versus a conventional dosage of zoledronic acid was estimated to be 2.309 (99% CI, 1.067-5.012) in patients with baseline serum NTx of more than 18 nM BCE, indicating a superiority of weekly low dose of zoledronic acid.

**Conclusions:**

ER, serum VEGF level after intervention, and numbers of chemotherapy regimens administered are prognostic but not predictive factors in breast cancer patients with bone metastases. Patients with baseline serum NTx of more than 18 nM BCE might benefit more from weekly low-dose of zoledronic acid.

**Trial registration:**

ClinicalTrials.gov unique identifier: ClinicalTrials.gov: NCT00524849

## Background

Breast cancer is the most frequent malignancy in women. Bone metastases play a crucial role in this cancer entity and are an important cause of disability and morbidity. 70% of women with advanced disease suffer from bone metastases [1]. The median survival of bone metastases is about 2 years, and survival may be prolonged with new treatment regimens [2]. Zoledronic acid (Zometa, Novartis) is the only and the most potent bisphosphonate indicated for the management of solid tumor with bone metastases [3,4]. While the use of zoledronic acid once every year is sufficient for the treatment of postmenopausal osteoporosis, dosing every 3-4 weeks has emerged as an appropriate established strategy for the prevention and management of bone metastases [5,6]. However, for maximizing its antitumor effects, the dosing schedule of zoledronic acid must be optimized [7]. Despite the completion of a randomized weekly low dose of zoledronic acid therapy study in breast cancer patients with bone metastases, no clear recommendation concerning its use outside of clinical studies can be given. Although metronomic low-dose zoledronic acid is more effective than the conventional regimen and generates sustained reductions in circulating VEGF and NTx levels, as well as stabilization of serum CA 15-3 levels, no significant benefit in survival after a relative short follow-up could be shown [8].

Several biomarkers detected by biochemical analysis, such as NTx, CEA, CA15-3, or VEGF, are found to be of prognostic value in breast cancer patients with bone metastases treated with zoledronic acid [7-12]. The prognostic value of VEGF, an endothelial-cell-specific mitogen and survival factor, has been studied extensively by immunohistochemical assay or enzyme-linked immunosorbent assay (ELISA) in various solid tumors. VEGF's status is an independent indicator of prognosis in most types of solid tumors. Over-expression of VEGF results in early relapse and poor survival. In addition, VEGF levels correlate with prognosis of breast cancer patients and intervention-induced reductions indicate a good prognosis [9,10]. NTx, a bone resorption marker correlates with both the presence and extent of bone metastases [11,12]. Elevated serum levels of NTx in the majority of patients with bone metastases can be normalized within 3 months of treatment of zoledronic acid [11]. Patients with a normalized NTx after treatment with zoledronic acid have a similar prognosis as those with a normal pretreatment NTx level, but a longer progression-free survival than those still with higher NTx levels after treatment [11,12]. Therefore, serum NTx level can be used to assess not only the inhibition of osteoclastic activity by bisphosphonates, but also the parameters of disease outcome. CEA and CA 15-3 are the most commonly used tumor markers. Use of CEA in conjunction with CA 15-3 improves the detection of systemic disease, while CA 15-3 itself is quite useful in metastatic bone breast cancer [13,14].

In the current study, we analyzed the prognostic and predictive value of these clinical and biochemical factors in 60 breast cancer patients with bone metastases who were treated within a randomized study (ClinicalTrials.gov number, NCT00524849) comparing weekly low-dose of zoledronic acid with conventional dose of zoledronic acid [8]. This is the first randomized study between weekly low-dose of zoledronic acid and conventional dose of zoledronic acid investigating the prognostic and predictive effect of clinical and biochemical markers.

## Methods

### Study design

#### Eligibility Criteria

Eligibility criteria included stage IV breast cancer with bone metastases. All patients had to be female; 18 years of age or older; with a performance status of 0, 1, or 2 on the Eastern Cooperative Oncology Group (ECOG) scale; a life expectancy of more than 3 months; and adequate organ function, including Cr ≤ 265 μmol/L, CrCl ≥ 30 mL/min, and Ca^2+ ^levels ranging from 2.0 mmol/L to 3.0 mmol/L. A negative pregnancy test was necessary for women in childbearing age. Diagnosis of bone metastases had to be made using X-ray, CT scan, or MRI. No anti-tumor therapy for stage IV breast cancer was permitted within 28 days before administration of the trial agent.

#### Exclusion Criteria

Patients were excluded from the study participation for any of the following reasons: (1) concomitant liver, brain, or symptomatic lung metastases (defined as hemoptysis, severe cough, and shortness of breath); (2) history of other malignancy, (unless more than 5 years disease-free) and excluding completely resected non-melanoma skin cancer; (3) active or uncontrolled infection; (4) concurrently active dental problems including infection of the teeth (maxillary or mandibular), dental trauma, or a concurrent or prior diagnosis of osteonecrosis of the jaw; (5) recent (within 6 weeks) or planned dental or jaw surgery; (6) history of uncontrolled or symptomatic angina, arrhythmias, or congestive heart failure; (7) previous treatment with any bisphosphonates within 1 month before study initiation; (8) known hypersensitivity to bisphosphonates; (9) history of treatment with calcitonin, gallium nitrate, or mithramycin within 14 days before study; or (10) pregnancy or lactation in potentially eligible women.

The study was a prospective randomized trial for advanced breast cancer patients with bone metastases to investigate the effects of two dose schedule of zoledronic acid. In the weekly low-dose arm, zoledronic acid (1 mg) was administered intravenously (IV) on days 1, 8, 15, and 22, followed by zoledronic acid (4 mg IV) on a standard 28-day schedule. In the conventional arm, zoledronic acid (4 mg IV) was administered every 4 weeks. The study was reviewed by the ethics committee of Fudan University Shanghai Cancer Center, and a written informed consent was obtained from all patients. The details of the study are described elsewhere [8].

### Blood samples

Blood samples of 60 patients treated within a randomized study comparing weekly low-dose of zoledronic acid with conventional dose of zoledronic acid between November 2006 and August 2008 were detected for the biochemical factors VEGF, NTx, CEA, and CA 15-3 to evaluate their prognostic and predictive effects. All the samples (4 samples per patient) were sent to Clinical Laboratory, Fudan University Shanghai Cancer Center, and stored for further biochemical analysis. No selection of the material was done and 228 specimens (228/240, 95%) were evaluable for analysis. For the remaining 12 time points (5 for weekly low-dose arm, 7 for conventional arm), no samples were available.

Peripheral venous blood samples (12 mL) were collected immediately (within 15 minutes) prior to zoledronic acid infusion on days 1, 15, 29, and 90. After collection, the samples were kept at room temperature (approximately 25°C) for 30 minutes to allow clotting, and were immediately (within 5 minutes) centrifuged at 1,000 × g for 15 minutes. Serum samples were aliquoted and stored at -80°C until assessment. White blood cell and platelet counts, hemoglobin, hepatic and renal function, serum electrolytes, and CEA and CA 15-3 levels were assessed at the time points mentioned above.

### Biochemical markers

Serum levels of VEGF were assayed using a solid-phase sandwich enzyme-linked immunosorbent assay (ELISA; Quantikine Immunoassays R&D Systems, France). The assay was performed in a single-blinded manner according to the manufacturer's instructions. Each sample was analyzed in duplicate. The minimum detectable level of VEGF was typically less than 9.0 pg/mL. The VEGF cut-off value was 500.0 pg/mL [15]. Corresponding platelet counts were also determined.

Serum levels of Osteomark NTx were assayed using a competitive-inhibition ELISA kit (Ostex International Inc., USA). NTx in the patient sample competed with the NTx epitope in the microplate well for antibody binding sites. The amount of bound labeled antibody was measured by colorimetric generation of a peroxide substrate at 450 nm. Assay values were reported as nanomoles Bone Collagen Equivalents per liter (nM BCE). The NTx cut-off value was 18 nM BCE [16].

Serum levels of CEA were assayed using electrochemiluminescence immunoassay -sandwich technique by cobas e immunoassay analyzers (Beckman Couter Inc., USA). Assay values were reported as micro gram per liter (μg/L). The range of normal value of CEA is 0-10 μg/L.

Serum levels of CA 15-3 were assayed using ELISA-sandwich technique by Access immunoassay system (Roche Diagnostics, USA). Assay values were reported as unit per milliliter (U/mL). The range of normal value of CA 15-3 is 0-25 U/mL.

### Statistical analysis

All statistical analyses were carried out on an intention-to-treat basis using the SPSS 15.0 software package (Chicago, IL, USA). The end point for all analyses was progression-free survival time (PFS). Progression-free survival time was defined as time from the date of randomization to the date of disease progression, or death from any cause. For patients who did not experience the event of interest during following up, the time from the date of randomization to the last documented follow-up was used as censored observation. The progression-free survival rates were calculated according to the Kaplan-Meier method. The hazard ratio between different groups defined by treatment or prognostic factors with corresponding confidence intervals was determined by the Cox regression model.

All data analyses were carried out according to a pre-specified analysis plan. The categorization of the prognostic factors--number of positive lymph nodes, tumor size, tumor grade, Her-2 status, estrogen receptor status, 2-year DFI (from surgery to first recurrence) - was predefined as used in the primary analysis and report of this study [8]. All biochemical analyses are based on all 60 patients and 228 samples. In a first step, the effects of the clinical and biochemical factors were analyzed in this patient population in a univariate analysis. Age, number of positive lymph nodes, estrogen receptor status, tumor size, tumor grade, Her-2 status, 2-year DFI (from surgery to first recurrence), numbers of chemotherapy regimens administered, interventions, baseline serum levels of VEGF, NTx, CEA, and CA 15-3, and VEGF levels at days 90 after intervention were included in a Cox regression model.. In a second step, a multivariate analysis of those clinical and biochemical factors was done, which had shown a significant effect in the univariate analyses at the 10% level.

For investigating the predictive effect of the clinical and biochemical factors, interactions between zoledronic acid treatment and the factors estrogen receptor status, number of positive lymph nodes, 2-year DFI, numbers of chemotherapy regimens administered, baseline serum levels of VEGF, NTx, CEA, and CA 15-3, and VEGF levels at days 90 after intervention were examined. After completing the univariate analyses, a multivariate analysis was done. Only variables with a significance of P < 0.05 in the univariate analyses were kept in the next Cox regression model. Multivariate analysis was done using a separate Cox regression model for each of these factors. The interaction was tested by a conditional LR test of equality of the treatment effects in the resulting groups. In virtue of multiple testing, a significance level of 1% was used for these tests. Additionally, the corresponding multiplicative interactive effects were estimated with 99% confidence intervals.

## Results

### Patient characteristics

For 60 patients, a complete data set of all investigated clinical and biochemical factor was evaluable. The clinical characteristics of the patients included in the current study are listed in Table 1. The 2 groups were well balanced with respect to their clinical and pathologic features. All patients received chemotherapy or hormonal therapy a minimum of 28 days after the first infusion of zoledronic acid, with no significant differences observed between the 2 arms in the use of anti-tumor agents. The details are described elsewhere [8].

**Table 1:** Patient characteristics

		Metronomic arm		Conventional arm		P
		No	%	No	%	
**Median age (range)**		46 (35-73)		51 (33-73)		0.1040
**ER status**	+	22	73.3	18	60.0	0.3950
	-	7	23.3	11	36.6	
**Her-2 status**	+	3	10.0	4	13.3	1.0000
	-	22	73.3	24	80.0	
**Primary tumor**	T1, T2	26	86.7	30	100.0	0.1120
	T3, T4	4	13.3	0	0	
Disease free interval*	>2 years	23	76.7	20	66.7	0.5670
	≤ 2 years	7	23.3	10	33.3	
**Lymph node**	0 ≤ N ≤ 3	25	83.3	22	73.3	0.5320
	N>3	5	16.7	8	26.7	
**Previous radiotherapy**	yes	18	60.0	20	66.7	0.7890
	no	12	40.0	10	33.3	
**Primary histology**	IDC	29	96.7	28	93.3	1.0000
	ILC	1	3.3	2	6.7	
**Grade**	1	9	30.0	6	20.0	0.7180
	2	14	46.7	16	53.3	
	3	4	13.3	3	10.0	
**Menopausal**	pre	17	56.7	14	46.7	0.6060
	post	13	43.3	16	53.3	
**Previous chemotherapy regimen**	≤ 2	26	86.7	22	73.3	0.3330
	>2	4	13.3	8	26.7	
**Bone metastasis sites**	1	11	36.7	9	30.0	0.7850
	≥ 2	19	63.3	21	70.0	

* Disease free interval = date from surgery to first recurrence

Table 2 shows the distribution of patients to the different categories of the biochemical factors.

**Table 2:** Biochemical factors investigated and categorizing

Biochemical factor	category		**No**.	%
**Baseline NTx**	normal	≤ 18 nM BCE	30	50
	abnormal	> 18 nM BCE	30	50
**Baseline VEGF**	normal	≤ 500 pg/ml	30	50
	abnormal	> 500 pg/ml	30	50
**Baseline CEA**	normal	≤ 10 μg/L	42	70
	abnormal	> 10 μg/L	18	30
**Baseline CA 15-3**	normal	≤ 51 U/mL	21	35
	abnormal	> 51 U/mL	39	65
**NTx (days 90)**	normal	≤ 18 nM BCE	34	56.7
	abnormal	> 18 nM BCE	16	43.3
**VEGF (days 90)**	normal	≤ 500 pg/ml	29	48.3
	abnormal	> 500 pg/ml	21	42.7

### Univariate analysis of prognostic clinical and biochemical factors

**Figure 1:**
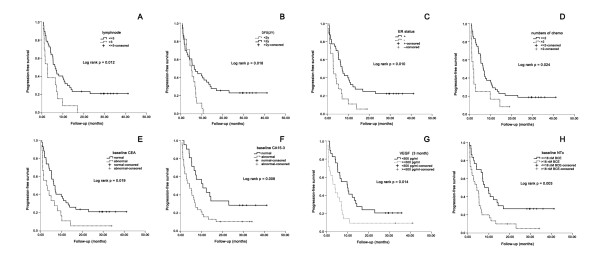
**Kaplan-Meier curves for progression-free survival with different biochemical and clinical factors**. A. Kaplan-Meier curves for progression-free survival. Notes: Patients with numbers of positive lymph nodes no more than 3 (N = 47), median = 6.3 months (95% CI, 4.1-8.6 months); patients with numbers of positive lymph nodes more than 3 (N = 13), median = 2.7 months (95% CI, 1.2-4.2 months); P = 0.012. B. Kaplan-Meier curves for progression-free survival. Notes: Patients with DFI no less than 2 years (N = 43), median = 6.4 months (95% CI, 2.9-10.0 months); patients with DFI less than 2 years (N = 13), median = 5.1 months (95% CI, 3.6-6.7 months); P = 0.018. C. Kaplan-Meier curves for progression-free survival. Notes: Patients with ER positive (N = 40), median = 7.0 months (95% CI, 5.3-8.8 months); patients with ER negative (N = 18), median = 2.8 months (95% CI, 2.2-3.4 months); P = 0.010. D. Kaplan-Meier curves for progression-free survival. Notes: Patients treated with 2 or less than 2 regimens (N = 48), median = 6.4 months (95%CI, 4.5-8.3 months); patients treated with more than 2 regimens (N = 12), median = 1.6 months (95%CI, 0.1-3.1 months); P = 0.024. E. Kaplan-Meier curves for progression-free survival. Notes: Patients with serum CEA baseline levels of less than 10 μg/L (N = 42), median = 6.4 months (95% CI, 4.6-8.3 months); patients with serum CEA baseline levels of greater than 10 μg/L (N = 18), median = 3.0 months (95% CI, 0-6.8 months); P = 0.019. F. Kaplan-Meier curves for progression-free survival. Notes: Patients with serum CA 15-3 baseline levels of less than 25 U/mL (N = 21), median = 10.6 months (95% CI, 3.5-17.8 months); patients with serum CA 15-3 baseline levels of greater than 25 U/mL (N = 38), median = 4.2 months (95% CI, 1.0-7.3 months); P = 0.008. G. Kaplan-Meier curves for progression-free survival. Notes: Patients with serum VEGF levels after 3 months of intervention of less than 500 pg/mL (N = 29), median = 10.0 months (95% CI, 4.3-15.7 months); patients with serum VEGF levels of 500 pg/mL or greater after 3 months of intervention (N = 21), median = 4.0 months (95% CI, 2.0-6.1 months); P = 0.014. H. Kaplan-Meier curves for progression-free survival. Notes: Patients with serum NTx baseline levels less than 18 nM BCE (N = 28), median = 8.1 months (95% CI, 4.2-11.9 months), patients with serum NTx baseline levels greater than 18 nM BCE (N = 32), median = 4.2 months (95% CI, 1.0-7.2 months); P = 0.003.

As the data updated [8], the median follow-up time was 31.4 months (ranges, 19.0-42.3 months). In univariate analyses of the clinical and biochemical factors, numbers of pretreated chemotherapy regimens, ER status, numbers of lymph nodes, 2-year DFI, serum VEGF level after 3 months of intervention, baseline serum CEA, CA 15-3, and NTx level showed a significant influence on the progression-free survival. (Figure 1)

### Multivariate analysis of prognostic clinical and biochemical factors

The results of a multivariate analysis of those clinical and biochemical factors at the 5% significance level in univariate analyses are displayed in Table 3. ER positivity, serum VEGF of less than 500 pg/mL after 3 months of intervention, baseline serum NTx of less than 18 nM BCE, and 2 or fewer chemotherapy regimens were associated with a significantly better PFS.

**Table 3:** Multivariate analysis of clinical and biochemical factors

Clinical and biochemical factor	category	HR (95% CI)	P
**Previous chemotherapy regimen**	≤ 2	1	0.000
	> 2	7.803 (2.884-21.112)	
**ER status**	-	1	0.001
	+	0.295 (0.141-0.618)	
**Baseline NTx**	≤ 18 nM BCE	1	0.001
	> 18 nM BCE	2.842 (1.458-5.539)	
**VEGF (days 90)**	≤ 500 pg/ml	1	0.020
	> 500 pg/ml	2.220 (1.136-4.338)	

### Analysis of predictive factors

**Figure 2:**
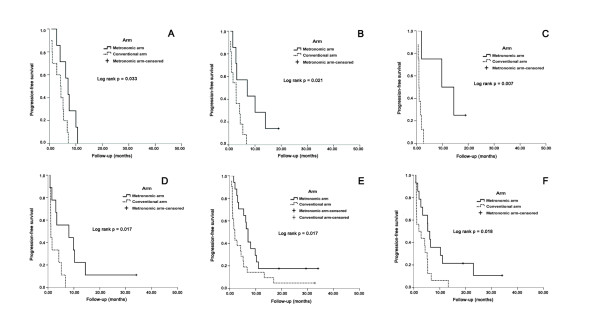
**Kaplan-Meier curves for progression-free survival with different treatments**. A. Kaplan-Meier curves for progression-free survival. Notes: Effect of treatment [weekly low-dose zoledronic acid (metronomic arm) versus conventional dose zoledronic acid (conventional arm)] in patients with DFI less than 2 years (N = 17), metronomic arm (N = 7), median = 7.0 months (95% CI, 4.7-9.3 months); conventional arm (N = 10), median = 4.2 months (95% CI, 1.7-6.6 months); P = 0.033. B. Kaplan-Meier curves for progression-free survival. Notes: Effect of treatment [weekly low-dose zoledronic acid (metronomic arm) versus conventional dose zoledronic acid (conventional arm)] in patients with ER negative (N = 18), metronomic arm (N = 7), median = 7.0 months (95% CI, 0-17.3 months); conventional arm (N = 11), median = 2.7 months (95% CI, 0.9-4.6 months); P = 0.021. C. Kaplan-Meier curves for progression-free survival. Notes: Effect of treatment [weekly low-dose zoledronic acid (metronomic arm) versus conventional dose zoledronic acid (conventional arm)] in patients with more than two of pretreated chemotherapy regimens (N = 12), metronomic arm (N = 4), median = 9.8 months (95% CI, 0-22.0 months); conventional arm (N = 8), median = 1.0 months (95% CI, 0.6-1.3 months); P = 0.007. D. Kaplan-Meier curves for progression-free survival. Notes: Effect of treatment [weekly low-dose zoledronic acid (metronomic arm) versus conventional dose zoledronic acid (conventional arm)] in patients with serum CEA baseline levels of greater than 10 μg/L (N = 18), metronomic arm (N = 9), median = 8.1 months (95% CI, 0-22.0 months); conventional arm (N = 9), median = 1.1 months (95% CI, 1.0-1.2 months); P = 0.017. E. Kaplan-Meier curves for progression-free survival. Notes: Effect of treatment [weekly low-dose zoledronic acid (metronomic arm) versus conventional dose zoledronic acid (conventional arm)] in patients with serum CA 15-3 baseline levels of greater than 25 U/mL (N = 38), metronomic arm (N = 17), median = 7.0 months (95% CI, 5.8-8.2 months); conventional arm (N = 21), median = 1.9 months (95% CI, 1.2-2.7 months); P = 0.017. F. Kaplan-Meier curves for progression-free survival. Notes: Effect of treatment [weekly low-dose zoledronic acid (metronomic arm) versus conventional dose zoledronic acid (conventional arm)] in patients with serum NTx baseline levels less than 18 nM BCE (N = 30), metronomic arm (N = 14), median = 5.4 months (95% CI, 3.8-7.0 months); conventional arm (N = 16), median = 1.9 months (95% CI, 0-5.3 months); P = 0.018.

Table 4 shows the results of the analyses regarding the predictive effect of the different clinical and biochemical factors (i.e., it was analyzed whether the treatment effect of weekly low-dose zoledronic acid versus conventional-dose zoledronic acid on progression free survival is heterogeneous in prognostic subgroups of patients defined by these factors). The results of univariate and multivariate analyses for patients undergoing Metronomic treatment are displayed in Table 5. Additionally, the progression-free survival in different subgroups is shown in Figure 2. In the univariate analyses, the weekly low-dose zoledronic acid seems to have a more pronounced effect in the subgroup with ER negativity, DFI less than 2 years, number of previous chemotherapy no less than two, serum CEA and CA 15-3 of greater than ULN, and with baseline serum NTx of greater than 18 nM BCE

**Table 4:** Subgroup analysis of PFS of patients treated with metronomic vs. conventional zoledronic acid

Clinical and biochemical factor		HR (95% CI)	P
**DFI (year)**	**> 2**	1.318 (0.660-2.630)	0.437
	**≤ 2**	3.455 (1.043-11.445)	0.043
**ER status**	**+**	1.036 (0.504-2.130)	0.922
	**-**	4.178 (1.130-15.450)	0.032
**Lymph node status**	**≤ 3**	1.340 (0.699-2.568)	0.378
	**> 3**	2.093 (0.636-6.893)	0.224
**Numbers of previous chemotherapy**	**≤ 2**	1.107 (0.586-2.093)	0.754
	**> 2**	11.440 (1.348-97.069)	0.025
**Baseline CEA (ug/L)**	**≤ 10**	1.281 (0.644-2.549)	0.481
	**> 10**	3.927 (1.179-13.079)	0.026
**Baseline CA 15-3 (U/mL)**	**≤ 25**	0.762 (0.258-2.251)	0.623
	**> 25**	2.087 (1.056-4.122)	0.034
**Baseline NTx (nM BCE)**	**≤ 18**	0.945 (0.399-2.241)	0.898
	**> 18**	2.520 (1.141-5.566)	0.022
**Baseline VEGF (pg/mL)**	**≤ 500**	2.337 (0.522-10.468)	0.267
	**> 500**	1.186 (0.546-2.573)	0.667
**VEGF (3 months after intervention) (pg/mL)**	**≤ 500**	0.834 (0.340-2.046)	0.692
	**> 500**	1.596 (0.619-4.115)	0.333

**Table 5:** Multivariate analysis of predictive factors for benefit from Metronomic treatment in patients undergoing metronomic zoledronic acid

Clinical and biochemical factor	Patient population	Multivariate			
		
		Variables in model		Backward selection	
		
		HR (99% CI)	P	HR (99% CI)	P
**DFI (year)**	**> 2 vs ≤ 2**	1.811 (0.788-4.161)	0.026	--	
**ER status**	**+ vs -**	0.753 (0.281-2.014)	0.459	--	
**Numbers of previous chemotherapy**	**≤ 2 vs > 2**	0.414 (0.130-1.136)	0.050	--	
**Baseline CEA (ug/L)**	**≤ 10 vs > 10**	1.633 (0.629-4.241)	0.186	--	
**Baseline CA 15-3 (U/mL)**	**≤ 25 vs > 25**	1.283 (0.433-3.800)	0.554	--	
**Baseline NTx (nM BCE)**	**≤ 18 vs > 18**	1.645 (0.652-4.150)	0.166	2.309 (1.067-5.012)	0.005

After multivariate analyses, baseline NTx level is the only factor for which a significant interactive effect with treatment on progression-free survival can be shown. The hazard ratio of weekly low-dose zoledronic acid versus conventional-dose zoledronic acid is estimated as 2.309 (99% confidence interval, 1.067-5.012) in high level of baseline NTx patients (> = 18.0 nM BCE), P = 0.005.

## Discussion

Several pilot studies have shown an advantage for weekly low-dose zoledronic acid in inducing an early significant and long-lasting decrease of VEGF levels in cancer patients [7,17]. Recently, after completion of a randomized study comparing weekly low-dose zoledronic acid with conventional-dose zoledronic acid in breast cancer patients with bone metastases, it has shown an advantage for weekly low-dose zoledronic acid. Low-dose zoledronic acid is shown to be more effective than the conventional regimen and generates sustained reductions in circulating VEGF and NTx levels, as well as stabilization of serum CA 15-3 levels [8]. The analysis presented in this article was undertaken to evaluate clinical or biochemical factors, which might predict the outcome after weekly low-dose or conventional-dose zoledronic acid in these breast cancer patients with bone metastases with poor prognostic factors; therefore, improve an individualization of the patient management.

In the first step of our analysis, we investigated the prognostic effect of the clinical and biochemical factors on progression-free survival in a patient population, which was treated in a randomized study protocol [8]. In a univariate analysis, after adjustment for treatment, clinical factors such as pretreated chemotherapy regimens, ER status, lymph nodes status, 2-year DFI, and biochemical factors such as serum VEGF level after 3 months of intervention, baseline CEA, CA 15-3, and NTx showed a statistically significant influence on progression-free survival. Patients with poor prognostic factors such as heavily treated with chemotherapy, ER negativity, more than 3 lymph nodes, DFI less than 2 years, high levels of CEA, and CA 15-3 had a significantly higher probability of relapse/death during follow-up, which is consistent with other reports [13,14,18-22]. For bone metastases and potential antiangiogenic effect of zoledronic acid, we added NTx and VEGF into analysis. We first claimed that patients with low level of baseline NTx and VEGF after intervention had a better prognosis than patients with high level of baseline NTx and VEGF after intervention tumors. No influence on progression free survival could be shown for other factors including age, tumor size, tumor grade, menopause, bone metastasis sites, baseline VEGF and NTx, CEA, and CA 15-3 levels after intervention.

In the multivariate analysis of the clinical and biochemical factors, numbers of pretreated chemotherapy regimens, ER status, baseline NTx, and serum VEGF level after 3 months of intervention were independent prognostic factors for progression free survival, showing that these factors were associated with a significant downward progression free survival. The results for numbers of pretreated chemotherapy regimens and ER status are in line with data of several other studies reporting a prognostic effect of these factors for breast cancer patients [18,20]. The result for the prognostic effect of NTx seems to be consistent with some studies in the literature. Several clinical studies suggest that the bone resorption marker, NTx, is associated with the presence and extent of metastases, response to treatment, and prognosis [23-25]. Arguably, the serum VEGF levels at 3-month time point, not the baseline serum VEGF levels was shown as the independent prognostic factor for PFS both in the univariate and multivariate analyses. Four published studies, which evaluated the prognostic value of serum VEGF level, revealed that high levels of circulating VEGF levels is associated with poor survival [15]. The explanation for prognostic value of serum VEGF level at 3-month time point could be that the VEGF values at that time reflect the dynamics of tumor biology interacting with zoledronic acid. However, this effect may need to be sustained with higher frequency and a longer duration of zoledronic acid administration.

The second aim of our study was to investigate whether the clinical or biochemical factors have predictive effects (i.e., if treatment effects of weekly low-dose zoledronic acid compared with conventional schedule are different in subgroups defined by the clinical or biochemical factors). This is the first study to investigate the predictive factors of breast cancer patients with bone metastases receiving weekly low-dose zoledronic acid. Several former studies have shown some predictive factors of conventional zoledronic acid or bisphosphonates naïve in bone metastases of several kinds of cancers. Lein et al. investigated in a study in 117 prostate cancer patients with bone metastases receiving zoledronic acid the predictive effect of a range of bone turnover markers, they created cox regression models with clinical factors and bone markers and eventually showed the baseline NTx level as predictor of SREs (HR, 3.33; 95%CI, 1.66-6.72, P = 0.0007) [26]. Coleman et al. investigated in three large, randomized trials of 1824 patients with bone metastases receiving bisphosphonates the prognostic effects of NTx, and bone alkaline phosphatase (BAP) and found that high NTx level in each solid tumor category were associated with a 4- to 6-fold increased risk of death compared with low NTx levels and BAP showed correlation with negative outcomes [11]. Brown et al. investigated two phase III randomized trials in 431 bisphosphonate-naïve patients with bone metastases secondary to prostate cancer, non-small-cell lung cancer, and other solid tumors from the predictive and prognostic effect of a range of factors. They found the predictive relationships for NTx and BAP levels for the NSCLC, prostate cancer, and other tumors. Their analysis showed that a high BAP level at baseline was associated with an increased risk of negative clinical outcomes compared to patients with low baseline BAP levels (RR, 1.49; 95% CI, 1.02 to 2.17, P = 0.041). Furthermore, it also revealed a statistically significant association between baseline NTx levels, time to a first skeletal related event (P = 0.026), risk of disease progression (P = 0.029) and death (P = 0.001) [12].

The major finding of our study was a predictive value of NTx on treatment strategies. Patients with high level of baseline NTx showed a higher progression-free survival with weekly low-dose zoledronic acid compared with conventional q-4 weeks regimen. Our results obtained for the patients with weekly low-dose zoledronic acid are different from other reports. First, several previous studies have evaluated some predictive or prognostic value of NTx level, but the treatment they used were old generation of bisphosphonates such as pamidronate and clodronate, which is not potent as zoledronic acid in anti-bone resorption and potential antitumor effects [23,25]. Several study use zoledronic acid, but NTx prognostic or predictive value was obtained from bisphosphonates naïve patients or patients receiving the conventional dose of zoledronic acid [11,12,26]. Secondly, the endpoint or evaluation indicators of amount of study which investigated the predictive effects of bone turnover markers. (i.e. NTx was SRE or progression of bone metastases) [11,16,26]. Although some studies found weekly low-dose zoledronic acid could induce serum VEGF level reduction, and had potential antitumor effects, they did not do the prognostic or predictive factors analysis [7,17]. As a lot of reports used urine NTx [11,12,23-26], we used serum NTx due to the precision of time points of sample collection because several biochemical markers such as VEGF in our previous study was collected concomitantly [8]. By doing this, we can make the study more manageable and decrease the impact of other factors like time, temperature, and storage, etc [27].

There are several strengths in our trial. First, it is a prospective and randomized trial to investigate the prognostic and predictive clinical or biochemical factors in patients with bone metastases as the bone turnover markers. NTx, especially, can be a predictive factor in cancer patients with bone metastases receiving conventional schedule of bisphosphonates. Our study further investigates the NTx predictive value in a new weekly schedule of low dose zoledronic acid. Secondly, recruitment of patients were strictly limited to breast cancer patients with bone metastases [8], thus the predictive effect of NTx may be helpful for the individualized therapy. High levels of baseline serum NTx may favor the weekly low-dose zoledronic acid; however, there are several drawbacks and limitation in our trial. First, weekly low-dose zoledronic acid was no longer given in the metronomic arm one month after the first drug administration. During the first month, the metronomic arm showed a significant reduction of VEGF and NTx compared with the conventional arm. This effect may need to be sustained, with higher frequency of zoledronic acid administration, over a longer time period. Second, this is a single center phase II trial. If the prognostic and predictive factors are analyzed from several randomized phase III, double blind studies, the results will be more credible. Thirdly, in our previous study, all patients received chemotherapy or hormonal therapy for a minimum of 28 days after the first infusion of zoledronic acid according to our institutional guidelines. No significant differences were observed between the 2 arms in the use of different agents due to a small sample size. This may potentially have impact on PFS. Finally, the overall survival results may be more credible for the evaluation of prognostic or predictive factors; the results will be further reported.

## Conclusions

We conclude that numbers of pretreated chemotherapy regimens, ER status, and baseline NTx and serum VEGF level after 3 months are prognostic factors in breast cancer patients with bone metastases receiving zoledronic acid. We firstly demonstrated that baseline NTx level had a predictive value on progression-free survival in patients treated with weekly low-dose of zoledronic acid, indicating that those with higher NTx level might benefit more from this new dosage. Our study maybe helpful for individualized therapy, however, further evaluation of the clinical and biochemical parameters of the weekly low-dose zoledronic acid regimen of randomized phase III study is warranted.

## List of abbreviations

VEGF: vascular endothelial growth factor; DFI: disease free interval; NTx N-telopeptide of type I collagen; CEA: Carcinoembryonic antigen; CA: 15-3 Carcinoma Antigen 15-3; ULN: Upper Limit of Normal; PFS: progression free survival; ER: estrogen receptor; HR: hazard ratio; CI: confidence interval; ELISA: enzyme-linked immunosorbent assay; IV: Intravenously; BAP: bone alkaline phosphatase; NSCLC: non-small cell lung cancer.

## Competing interests

The authors declare that they have no competing interests.

## Authors' contributions

XZ, QZ, JZ, SS, ZJ, BW, and ZW participated in acquisition of data, XZ, and XH participated in the analysis and interpretation of data, and drafting of the manuscript. XX carried out the biomarker detections. XH conceived of the study, participated in its design and coordination and revised the final manuscript. All authors have read and approved the final manuscript.

## Pre-publication history

The pre-publication history for this paper can be accessed here:

http://www.biomedcentral.com/1471-2407/11/403/prepub
